# An *emm*5 Group A Streptococcal Outbreak Among Workers in a Factory Manufacturing Telephone Accessories

**DOI:** 10.3389/fmicb.2017.01156

**Published:** 2017-06-21

**Authors:** Mingliang Chen, Wenqing Wang, Lihong Tu, Yaxu Zheng, Hao Pan, Gangyi Wang, Yanxin Chen, Xi Zhang, Linying Zhu, Jian Chen, Min Chen

**Affiliations:** ^1^Shanghai Municipal Center for Disease Control and PreventionShanghai, China; ^2^Shanghai Institutes of Preventive MedicineShanghai, China; ^3^Pudong New Area Center for Disease Control and PreventionShanghai, China

**Keywords:** *Streptococcus pyogenes*, *emm*5, factory, outbreak, pulsed-field gel electrophoresis (PFGE), whole genome sequence

## Abstract

Ranked among the top10 infectious causes of death worldwide, group A *Streptococcus* (GAS) causes small- and large-scale outbreaks, depending on the trigger as transmission of a GAS strain or expansion of predominant clones. In China, GAS infections other than scarlet fever are not notifiable. In Shanghai, an epidemiological investigation was initiated after two successive severe pneumonia cases with one death in a digital factory, from where outbreaks are less widely reported. The investigation was performed using *emm* typing, pulsed-field gel electrophoresis (PFGE) typing, superantigen profiling, and genome analysis. This enabled characterization of relatedness among the outbreak isolates and identification of the mobile genetic elements present. Among 57 patients with respiratory symptoms investigated in the factory, *emm*5 GAS strains were isolated from 8 patients. The eight GAS infection cases comprising one fatal severe pneumonia case, six influenza-like illness cases, and one pharyngitis case. Two risk factors were identified: adult with an age of 18–20 years and close contact with a GAS patient or carrier. GAS attack rate was 14.0% (8/57), and GAS carriage rate was probably around 2.7% (14/521) based on surveys in two nearby districts. All the 10 outbreak associated isolates were assigned to *emm*5 and sequence type ST-99 (*emm*5/ST-99), harbored superantigen genes *speC, speG*, and *smeZ*, and were assigned to two similar PFGE patterns (clones). Among the outbreak associated isolates, all carried *ermA* with resistance to erythromycin and inducible resistance to clindamycin, and eight (80%) carried a *tetM* gene with resistance to tetracycline. Among the 14 carriage isolates, 12 were *emm*12/ST-36, and 2 were *emm*1/ST-28, all with superantigen genes *speC, speG, ssa*, and *smeZ*. All the carriage isolates harbored *ermB* and *tetM* with resistance to erythromycin, clindamycin, and tetracycline. Genome analysis showed the two outbreak clones were closely related and possessed new prophages carrying virulence gene *sdc* and antibiotic resistance genes of *ermA* and *tetM*, which were not found in the *emm*5 reference strain Manfredo. This is the first report of a GAS outbreak in this type of workplace. The outbreak was caused by two closely related *emm*5 clones that differed from the predominant *emm* types circulating in China.

## Introduction

*Streptococcus pyogenes*, one of the top10 infectious causes of death worldwide (Excler and Kim, 2016), is also known as group A *Streptococcus* (GAS). GAS is responsible for at least 517,000 deaths each year (Carapetis et al., 2005). It can cause a broad spectrum of diseases, from superficial infections (e.g., pharyngitis and impetigo) to invasive infections (e.g., necrotizing fasciitis and streptococcal toxic shock syndrome), and to immune-mediated diseases (e.g., acute rheumatic fever and rheumatic heart disease).

Group A *Streptococcus* is well established as a cause of outbreaks, which can occur not only in a school, a military camp or a hospital but also throughout a city or even a country (Wasserzug et al., 2009; Chen et al., 2012; Dooling et al., 2013; Ben Zakour et al., 2015; Cornick et al., 2017). Small-scale GAS outbreaks can be caused by transmission of a GAS strain to vulnerable individuals, whether by person-to-person contact in crowded settings or from a common source (Walker et al., 2014). By means of recognizing the outbreak strain and comparing it with isolates from contacts and the environment, we can analyze the outbreak source, mode of transmission and risk factors; subsequently the appropriate prevention measures can be implemented (Raymond et al., 2005). Large-scale GAS outbreaks can occur with the emergence of predominant clones either as a result of the horizontal acquisition of DNA encoding toxins or antibiotic resistance determinants, or through more subtle genetic changes (Walker et al., 2014). We can take the ongoing outbreak of scarlet fever in China for an example. Scarlet fever, the only notifiable disease among all the GAS infections in China (Yang et al., 2013b), has been epidemic in Hong Kong and mainland China since 2011, with an incidence of 21.7–31.4/100,000 population (Lau et al., 2012; Yang et al., 2013b). This large outbreak is caused mainly by *emm*12 GAS, and typically infects children attending kindergarten and school (97%) (Chen et al., 2012). The original emergence of the scarlet fever *emm*12 clones in Hong Kong is thought to have been associated with acquisition of toxicity and multidrug resistance introduced by newly identified mobile genetic elements (MGEs) ICE-*emm*12 variants, ΦHKU.vir, and ΦHKU.ssa (Davies et al., 2015). The integrative and conjugative element (ICE) ICE-*emm*12 carries the macrolide-resistance gene *ermB* and tetracycline-resistance gene *tetM*; the prophage ΦHKU.vir encodes superantigens SSA and SpeC, and the DNase Spd1 (Tse et al., 2012); ΦHKU.ssa encodes superantigen SSA. Moreover, variants of ICE-*emm*12 and ΦHKU.vir were found to be transferred horizontally between *emm*1 and *emm*12 strains. This conferred enhanced virulence and multidrug resistance in *emm*1 strains and elevated their proportion in scarlet fever isolates (Ben Zakour et al., 2015). Against this background, it was considered appropriate to analyze whether the outbreak strains carry MGEs associated with toxin production and antimicrobial resistance.

Several molecular typing methods have been used to compare the outbreak strain with isolates from contacts and the environment in investigations of GAS outbreaks. One method is *emm* typing. The *emm* type has been used widely as an epidemiological marker in GAS surveillance and its distributions varied in high- and low-income countries (McMillan et al., 2013). The method is based on the variability of N-terminal region of the *emm* gene, the encoding gene of the matrix (M) protein which is a surface protein, vaccine antigen, and major virulence factor of GAS (McMillan et al., 2013). At least 248 *emm* types have been reported (Beall, 2014). Another method is pulsed-field gel electrophoresis (PFGE) typing, which has been considered to be among the “gold standard” tools used to investigate the clonal relatedness in epidemic surveys and to study outbreaks (Vitali et al., 2015). PFGE involves digesting the chromosomal DNA with restriction endonucleases that cleave infrequently, acquiring ‘fingerprint’ patterns by electrophoresis in pulsed field, and comparing the DNA restriction patterns of the isolates with one another to determine their relatedness (Tenover et al., 1995). The third method is superantigen profiling. Eleven superantigen-encoding genes have been reported in GAS, including three located in the chromosome (*speG, speJ*, and *smeZ*) and eight in prophages (*speA, speC, speH, speI, speK, speL, speM*, and *ssa*) (Sriskandan et al., 2007). They encode another important virulence factor of GAS: secreted pyrogenic exotoxin (SPE), which acts as a superantigen with pyrogenicity. It also has mitogenic activity for specific T cell subsets, which can increase host susceptibility to endotoxic shock and suppress immunoglobulin production (Commons et al., 2014). A single GAS strain usually harbors 4–6 superantigen genes, and these can provide a range of profiles for strain characterization. The last and the most powerful method is whole genome sequencing (WGS). On the one hand, phylogenetic analysis based on core genome genes (orthologous genes shared by all isolates) can disclose the relatedness between an outbreak strain and other isolates in the outbreak. On the other hand, genomic analysis can identify MGEs harbored in the GAS genome. The GAS genome is remarkable for its content of prophages, streptococcal phage-like chromosomal islands (SpyCIs), and other MGEs such as ICEs (Bessen et al., 2015).

This study is focused on the molecular investigation of a GAS outbreak in adults in a factory in China, where outbreaks are less widely reported. The investigation was triggered by the occurrence of two severe pneumonia cases in the factory during 9 days, including a fatal case. We not only characterized the relatedness among the outbreak strains and isolates from contacts but also identified the MGEs in the outbreak strains by means of *emm* typing, PFGE typing, superantigen profiling and whole genome analyzing. This report adds significantly to the limited data on adult GAS infections in China (Liu et al., 2014).

## Materials and Methods

### Study Setting

The factory producing mobile phone accessories is located at an industrial zone of Pudong New District of Shanghai. It covers an area of 520 acres, and employs over 70,000 workers aging from 16 to 45 years, who come from other provinces of China. The employees work and live in the industrial zone, where there are various facilities and shops. Over 3,000 workers work simultaneously on the same factory floor. Ten or twelve employees reside in a dormitory of about 28 square meters, sharing toilet facilities with other employees on the same floor. Locations where they work and live are centrally air-conditioned. There are seven departments in the manufactory, and each has a corresponding canteen equipped with an isolated kitchen. Only dining card consumption is accepted in all the seven canteens, and each employee has to consume in the assigned canteen corresponding to the department, for the card cannot be used in other canteens.

### Invasive Case Definition and Case Finding

A confirmed invasive GAS case was defined as a patient in whom the illness was ascribed to GAS that was isolated from a normally sterile site (e.g., blood and pleural effusion), while a probable invasive GAS case had the bacteria isolated from a non-sterile site (e.g., sputum and throat). To identify more cases, we reviewed all medical records and laboratory reports of patients with respiratory symptoms in the factory from January 1, 2012 to October 9, 2013. Active surveillance was conducted on patients with signs or symptoms of respiratory infection by collecting sputum and throat specimens from October 10 to October 14. Each specimen was inoculated onto Columbia sheep blood agar plate, chocolate blood agar plate, and MacConkey agar plate (Oxoid, Basingstoke, United Kingdom) in order to isolate *Staphylococcus aureus, S. pneumonia, S. pyogenes, Pseudomonas aeruginosa, Haemophilus influenza*, and *Klebsiella pneumoniae*. A standardized data-abstraction form was used to collect demographic and clinical information of each patient.

### Case-Control Study

To study the risk factors associated with GAS infection, including confirmed and probable cases, a case-control survey was performed. Control subjects were those patients in the factory with respiratory symptoms whose specimens were collected from October 10 to October 14 but without evidence of GAS colonization. We used a standardized data-abstraction form to obtain information including workshop, dormitory assignment, clinical presentation, medical and surgical histories. Statistical analysis was performed using SPSS (version 20.0; IBM), and statistical significance was assessed at *p* < 0.05.

### Close Contact Investigation and GAS Carriage Surveys

We screened for GAS colonization among close contacts, who were defined as persons with experience of working and living with a confirmed GAS case. Environmental samples swabbed from the filter mesh at the air outlet of air conditioners, quilt, door handle, and washstand in the patients dormitory in the working and living rooms of the cases were also collected for GAS culture. We intended to conduct a GAS carriage survey among the healthy workers, but this was rejected by the administrator of the factory. We then performed a survey in two randomly selected districts of Shanghai by culturing throat swabs from October 15 to December 20 of 2013. The survey covered five age groups (<6 years, 7–14, 15–19, 20–60, and >60 years). The age group < 6 years were pre-school children, mostly in kindergarten. The age groups 7–14 years and 15–19 years were students in different grades, while 20–60 years and >60 years comprised residents in the community. Specimens were obtained by swabbing the posterior pharynx with ESwab Collection Kits (Copan, Brescia, Italia) and were inoculated onto Columbia sheep blood agar at 36°C with 5% carbon dioxide. A carrier was defined as a healthy person without any epidemiological relationship with a GAS case, but with GAS cultured from a non-sterile site.

### Strain Identification and DNA Extraction

After culture for 24 h, β-hemolytic Gram positive cocci were tested by latex-agglutination with the Diagnostic Streptococcal Grouping Kit (Oxoid, Hampshire, United Kingdom). Isolates with the Lancefield group A antigen were identified by Vitek 2 system (bioMérieux, Marcy l′Etoile, France). Chromosomal DNA was extracted according to the protocol of Centers for Disease Control and Prevention^1^ (CDC).

### Antimicrobial Susceptibility Testing

The minimum inhibitory concentrations (MICs) of 12 antimicrobial agents including erythromycin, clindamycin, and tetracycline were determined by broth dilution method and interpreted by the breakpoints according to the guidelines of the Clinical and Laboratory Standards Institute (CLSI) in 2015 (CLSI, 2015).

### Detection of Resistance-Associated Genes and Mobile Genetic Elements

Macrolide resistance-related genes (*ermB, ermA*, and *mef*), tetracycline resistance-related genes (*tetM* and *tetO*), Tn916-Tn1545 transposon family (*intTn* and *xis*), and the novel MGEs ICE-*emm*12 and ΦHKU.vir, which were first identified in Hong Kong scarlet fever outbreaks in 2011, were screened by PCR as previously described (Perez-Trallero et al., 2007; Tse et al., 2012).

### Molecular Typing for GAS

All isolates were characterized by *emm* typing, multi-locus sequence typing (MLST), superantigen profiling, and PFGE typing with the restriction endonuclease *Sma*I (TaKaRa, Dalian, China) as previously described (Chen et al., 2012). PFGE images were analyzed with BioNumerics software package (version 6.0; Applied Maths, Austin, TX, United States) using the unweighted pair group method and an arithmetic averages (UPGMA) clustering algorithm. According to the Tenover criteria (Tenover et al., 1995), isolates with 0, 2–3, 4–6, and ≥7 bands different from the outbreak strain were designated, respectively, as genetically indistinguishable, closely related, possibly related, and different pattern. In other words, the isolates were part, probably part, possibly part, and not part of the outbreak, respectively. Isolates with genetically distinct PFGE pattern were assigned to different clusters.

### Genome Sequencing and Analysis

For Illumina pair-end sequencing of each strain, at least 3 μg genomic DNA was used for sequencing library construction as described previously (Harris et al., 2010). After a library quality test, Raw sequencing data was generated by Illumina base calling software CASAVA v1.8.2^2^ according to its corresponding manuscript. Sickle^3^ was applied to conduct reads data trimming with default parameters to get clean data in this study. We used SOAPdenovo^4^ (v2.01) to assemble genomes with multiple K-mer parameters and got the optimal results of the assembly. GapCloser software^4^ was subsequently applied to fill up the remaining local inner gaps and correct any single base polymorphism for the final assembly results. This *emm*5 Whole Genome Shotgun project has been deposited at GenBank under the accession NCTL00000000 and NCTM00000000. The versions described in this paper are NCTL01000000 and NCTM 01000000. The genome of Manfredo was set as a reference. Manfredo was the only *emm*5 strain genome present in GenBank (accession number AM295007) and was isolated from an acute rheumatic fever patient in 1952 in the United States (Holden et al., 2007). We also sequenced one *emm*1 isolate and three *emm*12 isolates in Shanghai, and performed the phylogenetic analysis by MEGA7 using the Neighbor-Joining method (Kumar et al., 2016). The accession numbers of the isolates involved in the phylogenetic analysis are shown in Supplementary Table S1.

Prophages were predicted using PHAST^5^ (Zhou et al., 2011). Superantigen-encoding genes (*speA, speC, speG, speH, speI, speJ, speK, speL, speM, ssa*, and *smeZ*), virulence genes (*spd3, sdc, sdaB, sdaD, speB, spyCEP, scpA, Mac*, and *sic*), antibiotic resistance-associated genes (*ermA, ermB, mef, tetK, tetL, tetM*, and *tetO*), serum opacity factor gene (*sof*), and streptococcal invasion locus (*sil*) were screened in the genomic sequences with the primers reported previously (Beall et al., 2000; Borek et al., 2012; Plainvert et al., 2014).

### Ethical Aspects

All specimens from patients and close contacts were collected as part of the routine clinical management of patients, according to the national guidelines in China. Consequently, informed consent was not sought from patients and close contacts, while informed consent was obtained from all participants in the carriage survey. The study was approved by Shanghai Municipal Center for Disease Control and Prevention ethical review committee (No.: 2016-4). All subjects gave written informed consent in accordance with the Declaration of Helsinki.

## Results

### Descriptive Epidemiology

From October 1 to October 9 of 2013, there were 42 cases of pneumonia in the factory. Two severe cases aged 18–20 years were transferred to a grade three hospital and one of them was dead on October 9. From then it was mandatory for the medical clinic of the factory to report the daily number of new cases, new cases with fever (>38°C), and transferred cases. During October 1 to October 20, the total number of cases with fever was 2,562, accounting for 15–25% of the daily new cases, and 1,836 cases were transferred to the grade three hospital, including 17 cases of severe pneumonia. Specimens including pleural effusion, sputum, and throat swab were collected from patients with respiratory symptoms in the factory on October 9 (the number of patients was six, *n* = 6), October 11 (*n* = 31), and October 14 (*n* = 20). The GAS attack rate was 14.0% (8/57), with nine isolates from eight confirmed cases including one confirmed invasive (the dead case, two isolates from pleural effusion and throat swab, respectively) and seven confirmed non-invasive cases (**Table 1**). The GAS case fatality rate was 12.5% (1/8). No other respiratory pathogenic bacteria including *S. aureus*,*S. pneumonia, P. aeruginosa, H. influenza*, and *K. pneumoniae* were detectable by culture.

**Table 1:** Clinical characterization of patients with *emm*5 group A *Streptococcus* (GAS) infection.

Case	Age, y/Gender	Date of symptom onset	Clinical diagnosis	Outcome	Source of GAS culture	*emm* subtype	Department
1	20/M	2013/10/2	Severe pneumonia, STSS^∗^	Died	Pleural effusion, throat swab	5.101	F4
2	27/M	2013/10/8	Influenza-like illness	Survived	Sputum	5.101	F3
3	20/M	2013/10/9	Influenza-like illness	Survived	Throat swab	5.101	F4
4	18/F	2013/10/10	Pharyngitis	Survived	Throat swab	5.101	F4
5	19/M	2013/10/13	Influenza-like illness	Survived	Throat swab	5.101	F5
6	20/F	2013/10/14	Influenza-like illness	Survived	Throat swab	5.101	F4
7	18/M	2013/10/14	Influenza-like illness	Survived	Throat swab	5.46	F3
8	18/M	2013/10/14	Influenza-like illness	Survived	Throat swab	5.101	F3

*^∗^STSS, streptococcal toxic shock syndrome.*

The fatal GAS case (Patient 1) was a 20-year-old man who was transferred to hospital by ambulance for septic shock on October 9th. About a week before, he had developed fever, anergy, cough, and expectoration under no obvious pre-disposing causes, with the signs of stethalgia, breathlessness, cardiopalmus, and oliguria (urinary volume less than 250 ml per day). On examination, his temperature was 40.0°C, blood pressure was 110/70 mmHg, pulse rate was 155 beats per minute, and respiratory rate was 47 per minute. Chest computed tomography (CT) scan showed double pneumonitis and pleural effusion. Although appropriate treatment was performed, the patient died on the day of transfer to the hospital. The other seven probable GAS cases appeared between 8th October and 14th October, most of them showed fever (6/7), sore throat (5/7), headache (5/7), and nasal discharge (5/7). They all eventually recovered. The eight GAS case patients were aged from 18 to 27 years, and worked in three different departments (**Table 1**).

Reviewing the medical records and microbiology laboratory reports of the factory from January to September in 2013, we did not find any evidence of GAS infection, but discovered another fatal case of severe pneumonia.

### Case-Control Study

All the 8 GAS patients and 49 control subjects who had respiratory symptoms but without GAS colonization were included. By univariable comparison of demographic and clinical characteristics, case patients were more likely than control patients to be adults of 18–20 years old and to have a roommate or teammate with GAS infection or colonization (**Table 2**).

**Table 2:** Univariate comparison of demographic and clinical characteristics of cases and controls.

Demographic and clinical characteristics	Cases,*N* = 8 *n* (%)	Control,*N* = 49 *n* (%)	*P-*value
Age (18–20 year)	7 (87.5)	13 (26.5)	<0.05
Male	6 (75)	40 (81.6)	0.64
Died during admission	1 (12.5)	0 (0)	0.14
Roommate or teammate with GAS colonization	2 (25)	0 (0)	<0.05
Work overtime (≥10 h per day)	8 (100)	49 (100)	1.00
**Signs and symptoms**			
Fever (≥38.0°C)	7 (87.5)	24 (49.0)	0.06
Cough	5 (62.5)	31 (63.3)	1.00
Pharyngalgia	5 (62.5)	33 (67.3)	1.00
Headache	5 (62.5)	21 (42.9)	0.45
Muscular stiffness	3 (37.5)	18 (36.7)	1.00
Anergy	6 (75)	23 (46.9)	0.25
Nasal discharge	6 (75)	17 (34.7)	0.05

### Close Contact Investigation

There were 11 roommates and 9 teammates of the dead case (Patient 1), among whom 4 close contacts developed respiratory symptoms and were administrated with antibiotics before the survey. We managed to collect throat specimens of 3 roommates of Patient 1 while the other 13 close contacts rejected requests. One close contact of Patient 1 was GAS culture-positive. Thirty-nine environmental samples were cultured and none was GAS-positive.

### Carriage Survey

In the GAS carriage surveys conducted in other two districts, 14 (2.7%) of 521 healthy people were positive. The age groups and their frequencies of GAS carriage were ≤6 years old (5.4%, 4/74), 7–14 years old (7.4%, 9/122), 15–25 years old (0), 26–59 years old (0), and ≥60 years old (0.8%, 1/120).

### Molecular Characterization of Outbreak Isolates

**FIGURE 1:**
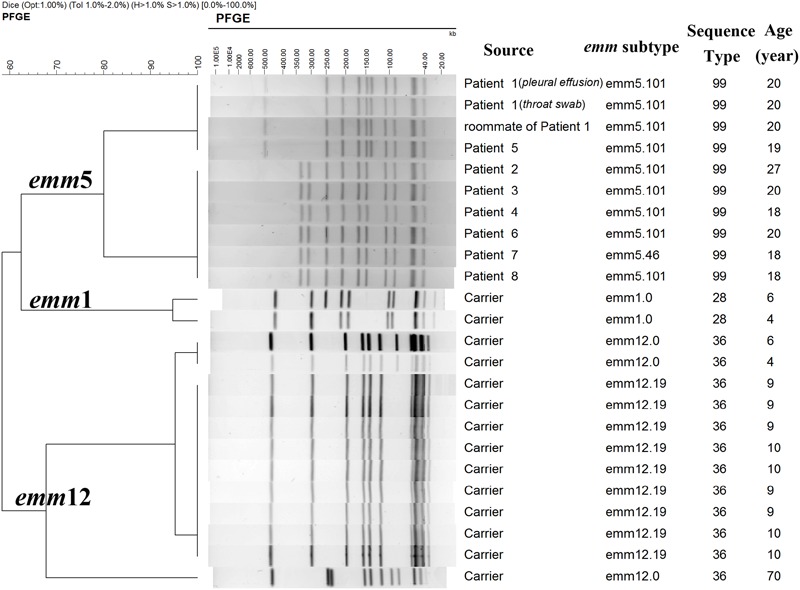
PFGE patterns of group A *Streptococcus* (GAS) isolates from patients and healthy people in the investigation. According to the Tenover criteria (Tenover et al., 1995), isolates with 0, 2–3, 4–6, and ≥7 bands different from the outbreak strain were designated as genetically indistinguishable, closely related, possible related, and different pattern, respectively, which means the isolates were part, probably part, possibly part, and not part of the outbreak, respectively. PFGE, pulsed-field gel electrophoresis.

All the 10 outbreak associated isolates, including nine isolates from eight cases and one isolate from a close contact, were assigned to *emm*5 and MLST profile of ST-99 (*emm*5/ST-99). There were two *emm* subtypes: 5.101 (*n* = 9; GenBank accession numbers as KJ807822-KJ807831) and 5.46 (*n* = 1), which differed by only one nucleotide. They all harbored superantigen genes *speC, speG*, and *smeZ.* The outbreak associated isolates were assigned to two possible related PFGE patterns (clones) with only four bands different (**Figure 1**). Isolates from Patient 1, his roommate and Patient 5 showed indistinguishable pattern, but there was no epidemiological relationship between the two patients. Isolates from the other six patients were also identified as indistinguishable, among which two patients were teammates. Screening of antimicrobial resistance-associated genes showed that all the outbreak associated isolates carried *ermA* corresponding to their resistance to erythromycin (4–>128 μg/ml), and inducible resistance to clindamycin (0.25–0.5 μg/ml, D-zone test positive), eight (80%) isolates carried *tetM* gene corresponding to their resistance to tetracycline (16–32 μg/ml). All the outbreak associated isolates harbored MGEs *intTn* and *xis* and none harbored ICE-*emm*12 or ΦHKU.vir.

### Molecular Characterization of Carriage Isolates

Among the 14 carriage isolates, which were isolated from asymptomatic carriers in the other two districts, 12 (86%) were assigned to *emm*12/ST-36, and 2 (14%) were *emm*1/ST-28. They all harbored *speC, speG, ssa*, and *smeZ*. Isolates of *emm*12 were assigned to three different PFGE patterns, among which two patterns differing in only one band were represented by 11 isolates (91.7%). The two *emm*1 carriage isolates were assigned to two PFGE patterns with one band different. All the carriage isolates harbored *ermB* and *tetM*, which was compatible with their resistance to erythromycin (>128 μg/ml), clindamycin (>128 μg/ml), and tetracycline (16–32 μg/ml). They all possessed MGEs *intTn, xis*, ICE-*emm*12, and ΦHKU.vir.

### Whole Genome Sequence Analysis

Based on the PFGE data, two *emm*5 isolates (spy0390 from pleural effusion of Patient 1 and spy0392 from sputum of Patient 2) were selected as the representatives of the two outbreak clones to perform genome sequencing. The assembled spy0390 draft genome was comprised of 1,953,330 bp with G+C content of 38.5%, and spy0392 of 1,934,710 bp with G+C content of 38.4%. Comparison was performed among the two assembled sequences and one *emm*5 reference genome sequence (Manfredo), the genome of which comprised 1,841,271 bp with a G+C content of 38.6%.

**FIGURE 2:**
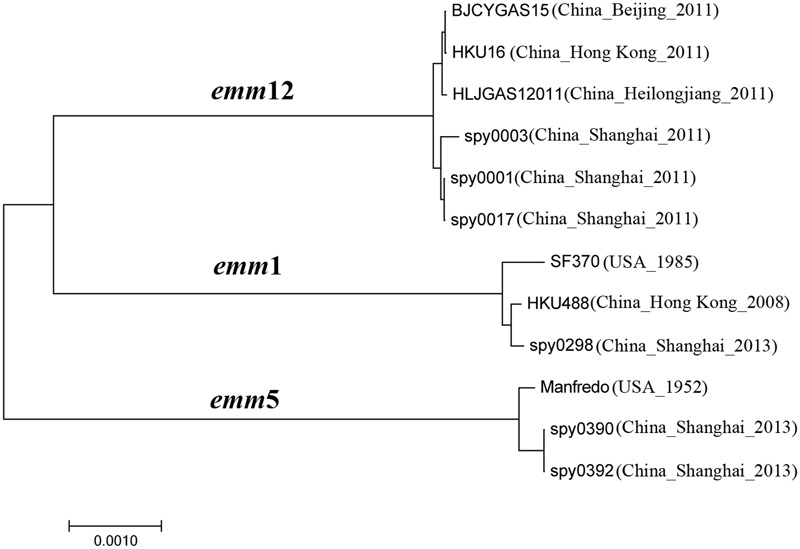
Phylogenetic analysis of *Streptococcus pyogenes* of *emm*1, *emm*5, and *emm*12 in Shanghai, China. The phylogenetic tree was generated in MEGA7 using the Neighbor-Joining method (Kumar et al., 2016). The optimal tree with the sum of branch length = 0.01685669 is shown. The tree is drawn to scale, with branch lengths in the same units as those of the evolutionary distances used to infer the phylogenetic tree. The evolutionary distances were computed using the *p*-distance method and are in the units of the number of amino acid differences per site (Nei and Kumar, 2000). All positions containing gaps and missing data were eliminated. There were a total of 421,361 positions in the final dataset.

Phylogenetic analysis was conducted with genomes of three *emm*5, three *emm*1, and six *emm*12 strains (**Figure 2**). There were three clades corresponding to each *emm* type. In the *emm*5 clade, outbreak isolates spy0390 and spy0392 showed a very close evolutionary relatedness and formed a distinct branch.

**FIGURE 3:**
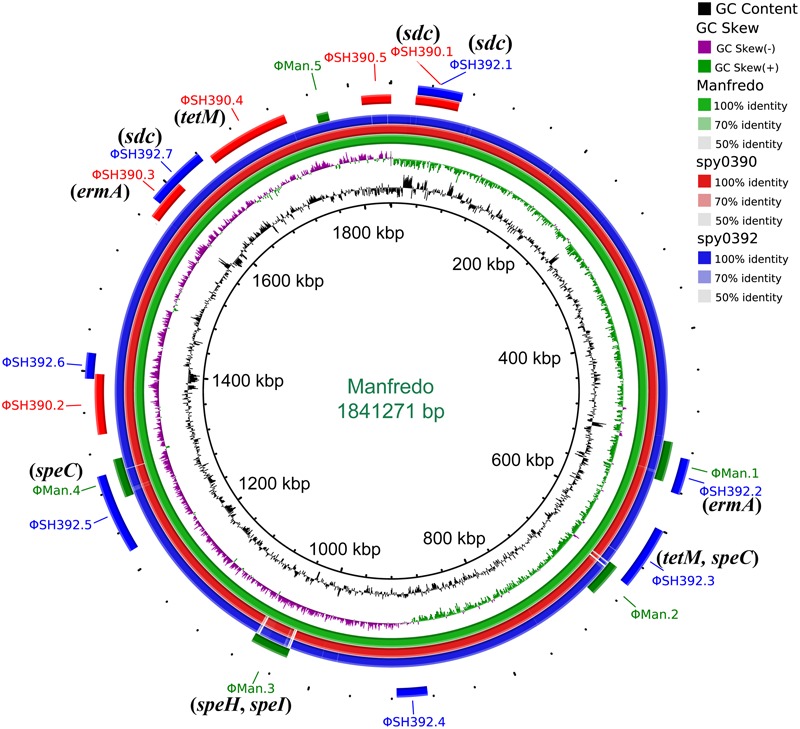
Circular genome map of *Streptococcus pyogenes emm*5 genome Manfredo with BLAST comparisons to the genomes of spy0390 and spy0392. The map was generated using BRIG (Alikhan et al., 2011). The innermost rings show G+C content (black) and G+C skew (purple/green) of Manfredo. The three outer rings show BLAST comparisons (using BLASTn and an *E*-value cutoff of 10.0) to the complete genome sequence of Manfredo (green) and the draft genome sequence of spy0390 (red) and spy0392 (blue). Legend shows percentage identity of BLASTn hits to the reference strain Manfredo. Labels around the outer ring refer to the insertion sites for predicted prophages with the corresponding color of the strain. Genes associated with antimicrobial resistance, superantigen, and virulence which are located within the prophages were labeled in parentheses.

The three *emm*5 genomes shared 1,646 predicted genes, including superantigen coding genes *speC, speG*, and *smeZ* and other virulence genes: *spd3, sdaB, speB, spyCEP, scpA, Mac*, and *sic*. The two outbreak isolates shared 292 more predicted genes with each other than with Manfredo, including virulence gene *sdc* and antibiotic resistance genes of *ermA* and *tetM*, all of which were located within the predicted prophages (**Figure 3**).

### Prophage Analysis of *emm*5

Five prophages were predicted to exist in spy0390 (ΦSH390.1 – ΦSH390.5), ranging in size from 41.9 to 84.1 kb, while seven prophages were predicted in spy0392 (ΦSH392.1 – ΦSH392.7), ranging from 25.1 to 78.1 kb (Supplementary Table S2). The macrolide-resistance gene *ermA* was located within the prophage ΦSH390.3 and ΦSH392.2, while tetracycline-resistance gene *tetM* was within ΦSH390.4 and ΦSH392.3 (**Figure 3**). The DNase coding gene *sdc* was present in the prophage ΦSH390.1, ΦSH392.1, and ΦSH392.7, therefore spy0392 possessed two copies of *sdc* (**Figure 3**). ΦSH390.1 and ΦSH392.1 were the same prophage, with the same insertion site and size. They shared 23.6% (10.3 kb/43.7 kb) nucleotide homology to ΦMan.3, which was one of the five prophages (ΦMan.1 – ΦMan.5) of Manfredo. The locations of ΦSH390.3 and ΦSH392.7 partially overlapped, but without any nucleotides in common. Other prophages in spy0390 and spy0392 had distinct locations but shared some associated nucleotides (Supplementary Table S2). ΦSH390.2 and ΦSH392.7 shared 38.2 kb nucleotides with Manfredo. ΦSH390.3 and ΦSH392.6 shared 13.2 kb nucleotides with Manfredo. ΦSH390.4 and ΦSH392.5 shared 17.2 kb nucleotides with Manfredo. ΦSH390.5 and ΦSH392.3 shared 19.5 kb nucleotides with Manfredo.

## Discussion

To the best of our knowledge, this is the first report of a GAS outbreak occurring in an electronic component factory in China. Due to the low labor cost, this type of factory is common in China. In this study, we identified two *emm*5 clones by PFGE which were responsible for the outbreak. They shared the same *emm* subtype (except one isolate with one nucleotide different), sequence type, superantigen profile, and resistance to macrolides. The phylogenetic analysis suggested the two clones were closely related.

Two risk factors associated with GAS infections were discovered. One risk factor was having a roommate or teammate colonized with GAS. The two clones were found transmitted between roommates and teammates, respectively. GAS can be transmitted by direct contact with respiratory secretions or skin lesions of GAS-colonized people (Deutscher et al., 2011). We supposed the person-to-person contact facilitated the spread of the outbreak clone. Therefore, the isolation of GAS cases may be an effective measure to prevent similar GAS infections. Another risk factor was age ranging from 18 to 20 years. It is interesting to find that 87.5% (7/8) of GAS patients were at ages between 18 and 20 years old; by contrast, the GAS carriage was low in this age group (0/120) outside the industrial zone. Taking into account that the workers were non-indigenous and seldom go outside, they could be considered to be closed populations, just like soldiers on military bases, patients in long-term care facilities, and students in schools, who were reported to be particularly vulnerable to GAS infection outbreaks (Wasserzug et al., 2009; Chen et al., 2012; Dooling et al., 2013). The factory had an intensive workload, poor-hygiene, and crowded conditions that might facilitate GAS outbreaks. In our investigation, typical factors pre-disposing to invasive GAS infection, such as old age, skin lesions, and underlying medical conditions (Steer et al., 2012), were not observed in the population of the factory.

The source of the outbreak was not discovered though we have cultured the environmental samples of the patients. The possibility of foodborne source could not be ruled out. Taking into account patients in this outbreak were from three departments (**Table 1**), and the rule that employees in different departments go to different canteens for meal, and the PFGE patterns were without correlation with the department of the patients, we supposed the outbreak less likely caused by food contamination.

Outbreaks of GAS infections involving *emm*5 are uncommon worldwide. In this outbreak, *emm*5 GAS caused a fatal case of invasive infection and several superficial infection cases, with a case fatality rate of 12.5%. Sporadic infections caused by *emm*5 GAS are relatively common and it was reported to be one of the 10-most prevalent *emm* types in Poland (15%), South Korea (4%), Ethiopia (2%), and Israel (2%) (Smeesters et al., 2009). In Europe, *emm*5 caused 2% of invasive infections, with a case fatality rate of 30% (Luca-Harari et al., 2009). A significant association of *emm*5 with pharyngitis and acute rheumatic fever has been reported (Walker et al., 2014). *emm*5 strains were estimated to be the 19th most common cause of all GAS diseases in Asia, causing about 2.5% of pharyngeal diseases (Steer et al., 2009). However, the prevalence of *emm*5 strains in China is currently unknown and this study provides the first report of *emm*5-causing GAS infection outbreaks in China.

The *emm*5 clones found in this study were characterized as being different from the predominant *emm* clones in China. During 2011–2012, epidemics of over 100,000 scarlet fever cases were reported in mainland China (Ben Zakour et al., 2015), with *emm*12 as the dominant clones (Chen et al., 2012; Yang et al., 2013b). Compared with the scarlet fever *emm*12 clones, the *emm*5 outbreak clones were deficient in superantigen gene *ssa*, which was supposed to be an important trigger of the expansion of the scarlet fever clones in Hong Kong (Davies et al., 2015). Besides, the *emm*5 clones harbored the macrolide-resistance gene *ermA*, while the scarlet fever *emm*12 clones harbored *ermB.* The different characterizations between the scarlet fever *emm*12 clones and the *emm*5 outbreak clones should probably be attributed to their harboring different MGEs. The scarlet fever *emm*12 clones have ICE-*emm*12 carrying *ermB* as well as ΦHKU.vir and/or ΦHKU.ssa encoding superantigen SSA (Tse et al., 2012; Davies et al., 2015), while the *emm*5 outbreak clones only have ΦSH390.3 or ΦSH392.2 to carry *ermA*, without other elements associated with SSA. On the other hand, although *tetM* and *speC* genes are also present in the *emm*5 outbreak clones, they are located within prophages different from those in *emm*12 strains.

By genomic analysis, we found that the outbreak isolates possessed the virulence gene *sdc* which was located within prophage but this was not found in the *emm*5 reference strain Manfredo. In *S. pyogenes*, the gene *sdc* (also termed sdalpha; Borek et al., 2011) encodes Streptococcal DNase α (*Sdα*). This was first identified in 2001 in a M3 *S. pyogenes* strain isolated from a toxic shock-like syndrome patient (Hasegawa et al., 2002). Although the function of *sdc* is not well understood, several studies on DNase proteins in *S. pyogenes* have shown their roles in facilitating GAS to thwart the host innate immune response, for example degrading neutrophil extracellular traps to promote neutrophil survival and suppressing Toll-like receptor 9-mediated innate immune responses and macrophage bactericidal activity (Buchanan et al., 2006; Uchiyama et al., 2012). The acquisition of *sdc* gene might enhance the ability of *emm*5 strains to colonize host cells.

A deficiency of this study is that we were unable to analyze the GAS carriage of the healthy population of the factory. The manager of the factory refused to authorize us to conduct the survey, for diseases caused by invasive GAS infections are not notifiable in China. Another deficiency of this study is that no food samples were collected throughout the investigation. Consumption of contaminated food can also cause outbreaks of GAS infections (Kemble et al., 2013; Yang et al., 2013a). Although indirect proof has decreased the possibility of foodborne source, it is necessary for us to collect direct proof to rule it out.

## Conclusion

We described an *emm*5 GAS outbreak in a factory making digital telephone components. There was substantial morbidity and mortality. The outbreak was caused by two closely related *emm*5 clones, which were characterized as being different from the predominant *emm* types circulating in China. The extent of the outbreak and the case fatality rate of 12.5% among GAS case patients should serve to alert the factory managers and public health experts to the higher risk of serious respiratory illnesses in this setting.

## Author Contributions

MC and JC conceived and designed the experiments. MLC, WW, and GW performed all experiments. HP, LZ, and XZ analyzed the data. LT, YC, and YZ assisted in antimicrobial susceptibility testing and molecular typing. YZ enrolled the patients. MLC and MC supervised the study and wrote the paper. All authors have read and approved the final manuscript.

## Conflict of Interest Statement

The authors declare that the research was conducted in the absence of any commercial or financial relationships that could be construed as a potential conflict of interest.

## Footnotes

1 https://www.cdc.gov/streplab/protocol-emm-type.html

2 https://support.illumina.com/sequencing/sequencing_software/casava.ilmn

3 https://github.com/najoshi/sickle

4 http://soap.genomics.org.cn/

5 http://phast.wishartlab.com/
