# Supplementary material for: An emm5 Group A Streptococcal Outbreak Among Workers in a Factory Manufacturing Telephone Accessories
Source: Front Microbiol. 2017 Jun 21;8:1156. doi: 10.3389/fmicb.2017.01156 (PMC5478724; doi:10.3389/fmicb.2017.01156)
Supplement: Supplementary file 1 [file Table_1.DOCX]

| T**able S1. Accession number of the genomes used in this study** | | | | | |
| --- | --- | --- | --- | --- | --- |
| Strain ID | Source | Year | *emm* type | Country (district) | Accession number |
| Manfredo | acute rheumatic fever | 1952 | 5 | USA | AM295007 |
| spy0390 | Severe pneumonia | 2013 | 5 | China (Shanghai) | NCTM00000000 |
| spy0392 | Influenza-like illness | 2013 | 5 | China (Shanghai) | NCTL00000000 |
| SF370 | wound | 1985 | 1 | USA | AE004092 |
| HKU488 | scarlet fever | 2008 | 1 | China (Hong Kong) | ERR172177 |
| spy0298 | scarlet fever | 2013 | 1 | China (Shanghai) | TBD* |
| spy0001 | carrier | 2011 | 12 | China (Shanghai) | TBD |
| spy0003 | close contact | 2011 | 12 | China (Shanghai) | TBD |
| spy0017 | scarlet fever | 2011 | 12 | China (Shanghai) | TBD |
| BJCYGAS15 | scarlet fever | 2011 | 12 | China (Beijing) | ALKD00000000 |
| HLJGAS12011 | scarlet fever | 2011 | 12 | China (Heilongjiang) | ALKE00000000 |
| HKU16 | scarlet fever | 2011 | 12 | China (Hong Kong) | AFRY01000001 |
| * TBD, to be determined | | | | | |
