# Supplementary material for: An emm5 Group A Streptococcal Outbreak Among Workers in a Factory Manufacturing Telephone Accessories
Source: Front Microbiol. 2017 Jun 21;8:1156. doi: 10.3389/fmicb.2017.01156 (PMC5478724; doi:10.3389/fmicb.2017.01156)
Supplement: Supplementary file 2 [file Table_2.DOCX]

**Table S2. Information of prophages in *emm5* isolates**

| Prophage ID | Length (kb) | Position (bp) | Gene associated with superantigen, antimicrobial resistance, or virulence | Most similar strain by blast (prophage) | Length and position of fragment in the most similar strain with 99% similarity (prophage) |
| --- | --- | --- | --- | --- | --- |
| ΦSH390.1 | 43.7 | 24,653 - 68,394 | *sdc** | Manfredo (ΦMan.3) | (1) 22.4kb , 34,333 - 56,723; (2) 7.2 kb, 1,058,058 - 1,065,662 (ΦMan.3); (3) 3.4kb, 30,944-34344; (4) 3.1kb, 1,054,769 - 1,057,889 (ΦMan.3) |
| ΦSH390.2 | 60.5 | 1,337,284 - 1,397,838 | */* | Manfredo | (1) 23.1kb, 302,476 - 325,531; (2) 15.2kb, 325,514 - 340,711 |
| ΦSH390.3 | 41.9 | 1,565,731 - 1,607,725 | *ermA** | Manfredo  (ΦMan.1 + ΦMan.4) | (1) 13.2kb, 513,122-526,322 (ΦMan.1); (2) 11.6kb, 525,881 - 537,042; (3) 4.0kb, 1,297,558 - 1,301,533 (ΦMan.4) |
| ΦSH390.4 | 84.1 | 1,648,642 - 1,732,804 | *spd3, tetM** | Manfredo (ΦMan.1 + ΦMan.3 + ΦMan.4) | (1) 17.2Kb, 1,038,044 - 1,055,414 (ΦMan.3); (2) 8.4kb, 537,039 - 545,409 (ΦMan.1); (3) 6.5kb, 549,883 - 556,422 (ΦMan.1); (4) 3.0kb, 1,300,836 - 1,303,895 (ΦMan.4); (5) 2.8kb, 1,279,620 - 1,276,762 (ΦMan.4) |
| ΦSH390.5 | 55.9 | 1,811,414 - 1,867,343 | */* | Manfredo  (ΦMan.2 + ΦMan.4) | (1) 19.5kb, 1,296,285 - 1,276,762 (ΦMan.4); (2) 16.1kb, 665,734 - 681,984 (ΦMan.2); (3) 7.0kb, 635,032 - 642,030; (4) 3.3kb, 682,107 - 685,371 (ΦMan.2) |
| ΦSH392.1 | 43.7 | 25,897 - 69,637 | *sdc** | Manfredo (ΦMan.3) | (1) 22.4kb, 34,333 - 56,723; (2) 7.2kb, 1,058,058 - 1,065,662 (ΦMan.3); (3) 3.4kb, 30,944 - 34,344; (4) 3.1kb, 1,054,769 - 1,057,889 (ΦMan.3) |
| ΦSH392.2 | 35.1 | 526,633 - 561,791 | *ermA** | Manfredo (ΦMan.1 + ΦMan.4) | (1) 11.2kb, 525,881 - 537,042 (ΦMan.1); (2) 10.7kb, 1,302,562 - 1,313,278 (ΦMan.4) |
| ΦSH392.3 | 62.2 | 599,941 - 662,209 | *speC, tetM** | Manfredo (ΦMan.1 + ΦMan.4) | (1) 19.5kb, 1,276,796 - 1,296,285 (ΦMan.4); (2) 13.2kb, 1,259,023 - 1,272,254; (3) 8.4kb, 537,039 - 545,409 (ΦMan.1); (4) 2.8kb, 1,276,762 - 1,279,620 (ΦMan.4) |
| ΦSH392.4 | 29.8 | 885,402 - 915,295 | */* | Streptococcus pyogenes strain STAB10015** | 16.7kb, 994,187-1,011,516 |
| ΦSH392.5 | 78.1 | 1,219,432 - 1,297,539 | */* | Manfredo (ΦMan.2+ΦMan.3+ΦMan.4) | (1) 19.5kb, 665,734 - 685,371 (ΦMan.2); (2) 17.2kb, 1,038,044 - 1,055,414 (ΦMan.3); (3) 7.7kb, 684,913 - 692,627 (ΦMan.2); (4) 4.0kb, 1,297,558 - 1,301,533 (ΦMan.4); (5) 4.0kb, 651,810 - 655,787 (ΦMan.2) |
| ΦSH392.6 | 25.1 | 1,392,765 - 1,417,936 | *spd3* | Manfredo (ΦMan.1) | (1)16.5kb, 509,864 - 526,322 (ΦMan.1); (2) 6.4kb, 549,883 - 556,295 (ΦMan.1) |
| ΦSH392.7 | 60.5 | 1,580,459 - 1,640,968 | *sdc** | Manfredo | (1) 23.0kb, 302,489 - 325,531; (2) 15.2kb, 325,514 - 340,724 |
| * gene not found in prophages of Manfredo; ** STAB10015, a strain assigned to *emm28* with GenBank accession number CP011068.1. | | | | | |
